# Molecular Survey of Hemopathogens in Bats from the Western Brazilian Amazon

**DOI:** 10.3390/pathogens14060527

**Published:** 2025-05-25

**Authors:** Abraão Isaque da Silva, Eliz Oliveira Franco, Ana Cláudia Calchi, Francisco Chagas Bezerra dos Santos, Rair de Sousa Verde, Victória Valente Califre de Mello, Daniel Antônio Braga Lee, Caroline Tostes Secato, Clara Morato Dias, Rosangela Zacarias Machado, André Luiz Rodrigues Roque, Marcos Rogério André

**Affiliations:** 1 Vector-Borne Bioagents Laboratory (VBBL), Department of Pathology, Reproduction and One Health, School of Agrarian and Veterinary Sciences, São Paulo State University, Jaboticabal CEP 14884-900, Brazil; abraao.isaque@unesp.br (A.I.d.S.);; 2 Laboratory of Trypanosomatid Biology, Instituto Oswaldo Cruz (FIOCRUZ), Rio de Janeiro CEP 21040-900, Brazil; 3 Graduate Program in Health and Sustainable Animal Production in the Amazon, Federal University of Acre, Rio Branco CEP 69920-900, Brazil

**Keywords:** anaplasmataceae, chiroptera, hemoplasmas, *Hepatozoon* spp., piroplasmids

## Abstract

Bats are considered reservoirs of several emerging zoonotic pathogens. Previous studies on blood parasites such as Anaplasmataceae, hemoplasmas, piroplasmids, and *Hepatozoon* spp. in South American bats have revealed high genetic diversity. This study investigated the molecular occurrence of these agents in 278 bats of 32 species captured in the state of Acre in the Western Brazilian Amazon. Spleen DNA samples were screened by PCR for different pathogens and tested negative for *Anaplasma* spp., *Ehrlichia* spp., *Neorickettsia* spp., piroplasmids, and *Hepatozoon* spp. However, 84 of the 208 samples (40.4%) were positive for hemoplasmas based on the 16S rRNA gene, and 15 out of the 84 (17.85%) were positive for the 23S rRNA gene. Seventeen 16S rRNA sequences, corresponding to 12 genotypes, were grouped with hemotropic *Mycoplasma* sp. previously detected in bats from Brazil, Peru, and Belize. Three 23S rRNA sequences represent three distinct genotypes clustered with hemotropic *Mycoplasma* sp., previously detected in *Desmodus rotundus*. This is the first molecular report of hemoplasmas in six bat species, namely *Dermanura cinereus*, *Lophostoma silviculum*, *Phyllostomus elongatus*, *Phyllostomus hastatus*, *Rhinophylla fischerae*, and *Sturnira tildae*.

## 1. Introduction

The order Chiroptera represents the second most diverse group of mammals described in Brazil, composed of 9 families, 68 genera, and 186 species identified in different regions of the country [1,2]. These mammals play an important role in the recovery and maintenance of forest environments, pollination, control of insect populations, and maintenance of the balance and functionality of ecosystem dynamics [3]. Due to the unique characteristics of the life history, such as long life expectancy (average of 10 to 20 years), large population densities, roosting behavior, and ability to fly, bats are considered ideal reservoirs for several emerging zoonotic pathogens [4].

The genera *Anaplasma*, *Ehrlichia*, and *Neorickettsia* (Rickettsiales: Anaplasmataceae) encompass obligate intracellular alphaproteobacteria capable of infecting a wide variety of animal and human blood cells [5,6]. While *Ehrlichia* and *Anaplasma* species are transmitted by Ixodida ticks [7], *Neorickettsia* spp. are transmitted to vertebrates through the ingestion of larval forms of digenetic trematodes infected by the agent [8,9]. Although such agents have been detected in bats and associated ectoparasites or trematodes in South America [10,11,12,13] and Europe [14,15,16,17], few studies have been conducted in Brazil [18,19]. In this sense, Ikeda et al. [20] detected that *Ehrlichia* spp. were phylogenetically associated with *Ehrlichia minasensis*, *Ehrlichia ruminantium*, and *Anaplasma* spp., related to *A. phagocytophilum* and *Neorickettsia* spp. in non-hematophagous bats and associated ectoparasites in Central–Western Brazil. Recently, *Ehrlichia* spp. phylogenetically related to *E. minasensis*, *Anaplasma marginale*, and *Neorickettsia* spp. phylogenetically associated with *N. risticii* that have been detected in hematophagous bats in the northern region of Brazil [19].

Hemoplasmas (Mycoplasmatales: Mycoplasmataceae) are epi-erythrocytic Gram-negative bacteria that can cause hemolytic anemia in animals and humans [21]. Although these bacteria have been reported in bats in several Brazilian states [22,23,24,25], the genetic diversity of such detected agents seems higher than previously noticed. The incrimination of bats of the Pteromalidae family in New Caledonia as reservoirs of ‘*Candidatus* Mycoplasma haematohominis’, a species of hemoplasma that causes clinical disease in humans [26], reinforced the need to investigate the diversity of hemoplasmas in bats.

Although piroplasmids (Piroplasmorida: Babesiidae/Theileriidae) have been extensively investigated in mammals worldwide [27], few studies have been conducted to detect these tick-borne apicomplex protozoa in Neotropical bats. *Babesia vesperuginis* has been pointed out as the main piroplasmid species detected in bats from Europe and Asia [28,29,30,31,32,33,34,35]. However, little is known regarding the molecular identity of piroplasmids in bats from other continents. A supposed novel *Babesia* sp. related to the *Babesia microti* group has been described in bats from Madagascar [36]. In South America, *Babesia* sp. was found in blood smears from bats sampled in Colombia [37]. At the same time, a putative novel Piroplasmorida species was molecularly detected in *Phyllostomus discolor* in Central–Western Brazil [38]. In contrast, *Babesia* spp. and *Theileria* spp., phylogenetically grouped in the clades “South American Marsupialia”, “*Theileria* sensu stricto”, and “*Tapirus terrestris*”, have been detected in hematophagous bats sampled in the northern region of Brazil [24].

*Hepatozoon* spp. (Adeleorina: Hepatozoidae) are apicomplexan protozoa transmitted by ingesting definitive invertebrate hosts (ticks, mosquitoes, flies, fleas, lice) and predation [39,40,41,42,43]. Few studies have reported *Hepatozoon* spp. in bats worldwide. So far, *Hepatozoon* spp. has only been detected in bats from Borneo [44] and Brazil [45,46]. The 18S rRNA sequences detected by *Hepatozoon* spp. were positioned in the same clade as *Hepatozoon* spp., detected in reptiles, rodents, amphibians, and marsupials [44,45,46].

Considering that Brazil has a huge bat fauna and plays a potential role in the maintenance and transmission of distinct hemopathogens worldwide, the present study aimed to investigate the occurrence and molecular identity of *Anaplasmataceae*, hemoplasmas, piroplasmids, and *Hepatozoon* spp. agents in non-hematophagous bats sampled in the state of Acre, in the Western Brazilian Amazon Forest.

## 2. Materials and Methods

### 2.1. Ethical Statement

The bat captures were approved by the Chico Mendes Institute for the Conservation of Brazilian Biodiversity (ICMBio—SISBIO), licenses 44089-1 and 47377-1. All procedures carried out followed protocols approved by the Ethics Committee on the Use of Animals of FIOCRUZ (LW81-12) and by the Ethics Committee on the Use of Animals of the Faculty of Agrarian and Veterinary Sciences of UNESP (CEUA FCAV/UNESP 3911/23).

### 2.2. Sampling and Study Area

**Figure 1:**
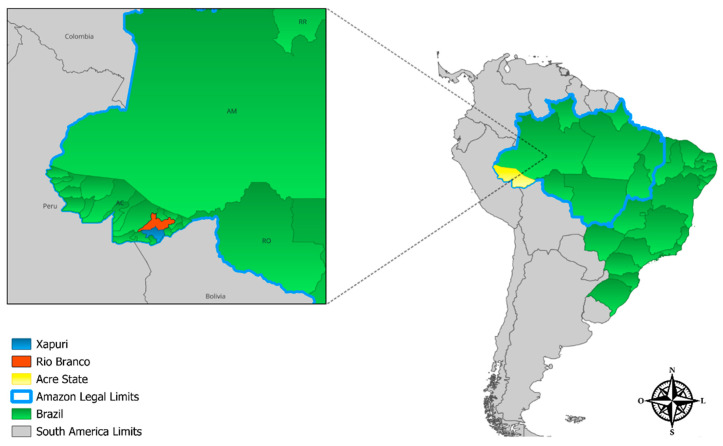
Representative image of Brazil (highlighted in green in South America) and location of the municipalities of Rio Branco (in orange) and Xapuri (in blue), state of Acre (in yellow), where the bats were sampled. QGIS Geographic Information System. Open Source Geospatial Foundation Project. http://qgis.osgeo.org (Accessed on 29 March 2025).

Between 2014 and 2015, 367 non-hematophagous bats were captured in two municipalities in the state of Acre: Rio Branco (09°58′29″ S/67°48′36″ W) and Xapuri (10°10′95″ S/68°30′16″ W) [47] (Figure 1). Morphological characters were used to identify the bats [48], which were classified into 23 genera and 30 species. Of the 367 bats captured [47], 278 of those for which spleen samples were available were included in the present study. Of this total, 196 samples were collected in Rio Branco and 82 in Xapuri (Table 1).

### 2.3. DNA Extraction and PCR for the Endogenous Mammalian Gene

DNA extraction from bat spleen samples was performed using the BioPur Mini Spin Plus Extraction Kit (Mobius Life Science, Pinhais, PR, Brazil), following the manufacturer’s instructions. After DNA extraction, the concentration and quality of the DNA (260/280 ratio) were evaluated using a spectrophotometer (Nanodrop, Thermo Fisher Scientific, San Jose, CA, USA). To verify the presence of amplifiable DNA and the absence of PCR inhibitors in the extracted samples, a conventional PCR assay targeting the endogenous mammalian glyceraldehyde-3-phosphate dehydrogenase (*gapdh*) gene was performed [50], as previously described [51]. Only positive samples for these assays were used in other PCR reactions to detect the infections.

### 2.4. PCR Tests for Screening and Characterisation of Haemopathogens

PCR assays targeting the 16S rRNA gene of *Anaplasma* spp. (548 bp) [52], *Neorickettsia* spp. (529 bp) [53] and hemoplasmas (620 bp) [54], the *dsb gene* of *Ehrlichia* spp. (409 bp) [55], the 18S rRNA gene of piroplasmids (800 bp) [56], *Hepatozoon* spp. (800 bp) [57], and (600 bp) [58] were performed. In addition, PCR assays based on the 16S rRNA (1380 bp) [54] and 23S rRNA (800 bp) [59] genes were conducted for further molecular characterization of the hemoplasma-positive samples.

Each PCR reaction had a total volume of 25 µL, containing 10× PCR buffer (Promega^®^, Madison, WI, USA), sterilized ultrapure water (Invitrogen^®^, Carlsbad, CA, USA), 0.2 mM of each deoxynucleotide, 0.4 µM of each oligonucleotide, 3.0 mM of MgCl_2_, 1.25 U Go Taq Hot Start Polymerase (Promega^®^, Madison, WI, USA), and 3 µL of DNA template. In nPCR assays for piroplasmids and *Neorickettsia* spp., 1 µL of the amplified product of the first PCR reaction was used as the target DNA in the second reaction. DNA samples of *Ehrlichia* spp., obtained from bats [19], hemotropic *Mycoplasma* spp., obtained from *Tapirus terrestris* [60], *Babesia vogeli* (Jaboticabal strain) [61], and *Hepatozoon* sp. detected in *Cerdocyon thous* [62] were used as positive controls. gBlocks^®^ (Integrated DNA Technologies, Coralville, IA, USA) containing an insert of the target fragments of the 16S rRNA gene from *Anaplasma phagocytophilum* and *Neorickettsia risticii* were also used as positive controls. Sterile ultrapure water (Invitrogen^®^, Carlsbad, CA, USA) was used as a negative control in all PCR assays.

### 2.5. Sequencing and Phylogenetic Analysis

The PCR products were purified using the ExoSAP-IT™ PCR Product Cleaning Reagent (Thermo Scientific, San Jose, CA, USA). Sequencing of the amplified products was performed using an automated technique based on the Sanger dideoxynucleotide chain termination method [63] in an ABI PRISM 3700 (Applied Biosystems, Waltham, MA, USA) DNA analyzer in the Human Genome and Stem Cell Research Center, Institute of Biosciences, University of São Paulo (USP), São Paulo, SP, Brazil.

An analysis of the electropherograms generated in the sequencing was performed, observing the quality of the peaks corresponding to each sequenced base using Bioedit v. 7.0.5.3 software [64]. To construct the consensus sequences, the Phred-Phrap version 23 [65,66] program was used, analyzing the “forward” and “reverse” strands sequenced from the same sample, respecting the minimum quality value of 20 for each nucleotide to determine the nucleotide sequence. The consensus sequences were subjected to analysis by BLASTn [67] for comparison with those deposited in GenBank “http://www.ncbi.nlm.nih.gov/genbank (Accessed on 25 February 2025)”.

Sequences saved in “FASTA” mode were aligned with other homologous sequences of the same sequenced gene retrieved from the GenBank database using MAFFT-Multiple Sequence Alignment Software version 7.0 available online “https://mafft.cbrc.jp/alignment/server/index.html” (Accessed on 25 February 2025) [68]. The phylogenetic analysis of maximum likelihood was performed using the W-IQ-Tree software available online “http://iqtree.cibiv.univie.ac.at/” (Accessed on 25 February 2025) [69,70]. Clade support for maximum likelihood analysis was evaluated using bootstrap analysis [71] with 1000 replicates. Phylogenetic tree editing, as well as rooting (via external group), was performed using the Treegraph 2.13.0 beta software [72].

### 2.6. Analysis of Genetic Diversity

The analysis of genetic diversity was performed using the sequences obtained in the analysis of the hemoplasm 16S rRNA and 23S rRNA. The calculation of nucleotide diversity (π), polymorphism level (haplotype diversity—[dh]), number of haplotypes (h), and the average number of nucleotide differences (K) between the sequences obtained was performed using the DnaSP v5 software [73].

## 3. Results

In total, 208 (74.8%) spleen samples were positive for the endogenous *gapdh* gene and submitted to diagnostic PCR reactions. All bats were negative in molecular screening assays targeting *Anaplasma* spp. 16S rRNA, *Ehrlichia* spp. *dsb*, *Neorickettsia* spp. 16S rRNA, piroplasmid 18S rRNA, and *Hepatozoon* spp. 18S rRNA. On the other hand, 84/208 (40.4%) of the bats were positive in PCR directed to the 16S rRNA gene of hemoplasmas (~620 bp), comprising different species of bats. Of these, 15/84 (17.85%) were positive in the molecular characterization based on the 23S rRNA gene (800 bp) (Table 2).

We were able to retrieve 20 high-quality consensus sequences: 17 for the 16S rRNA genes and 3 for the 23S rRNA genes. The number of sequences obtained, as well as the results of the BLASTn analysis, including the target gene, the coverage of the consultation, the E-value, and the percentage of identity, are described in Table 3.

**Figure 2:**
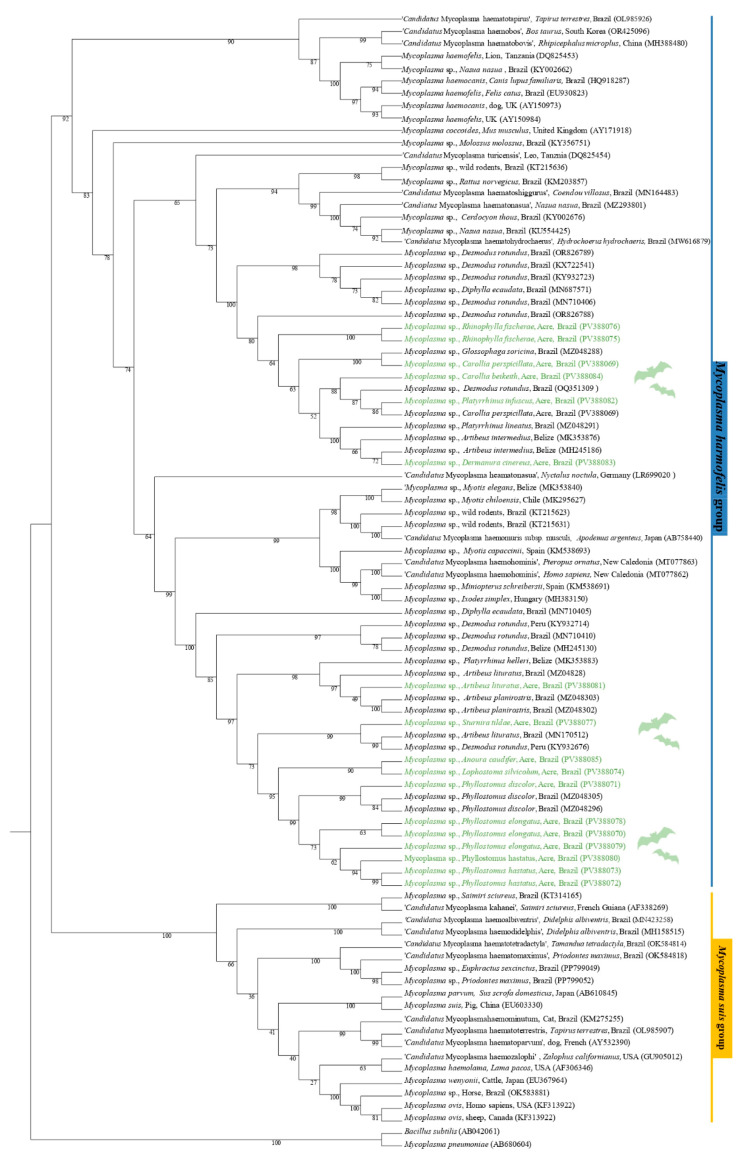
Phylogenetic tree based on a sequence alignment of the 16S rRNA gene (620 bp) of *Mycoplasma* spp., using the Maximum Likelihood (ML) method and GTR + F + I + G4 as an evolutionary model. The sequences detected in the present study are highlighted in green. *Mycoplasma pneumoniae* (AB680604) and *Bacillus subtilis* (AB042061) were used as outgroups.

The phylogenetic analysis based on the 16S rRNA gene (620 bp alignment) inferred by the Maximum Likelihood method and using GTR + F + I + G4 as an evolutionary model, grouped the sequences obtained PV388069 (*C. perspicillata*), PV388082 (*P. infuscus*), and PV388084 (*C. beikeith c.f.*) in the same clade with a sequence of *Mycoplasma* spp., detected in *D. rotundus* in Brazil, with bootstrap clade support of 88. In addition, the sequences PV388075 (*R. fischerae*) and PV388076 (*R. fischerae*) were positioned in the same clade, sister to the clade containing the PV388069 sequence and the *G. soricina* sequence from Brazil, supported by a bootstrap value of 100. The PV388083 sequence (*D. cinereus*) was positioned in a sister group with *Mycoplasma* sp. *sequences* previously detected in *Artibeus lituratus* from Belize and *Platyrrhinus lineatus* from Brazil, supported by a bootstrap value of 100. The sequences PV388070 (*P. elongatus*), PV388071 (*P. discolor*), PV388072 (*P. hastatus*), PV388073 (*P. hastatus*), PV388074 (*L. silviculum*), PV388078 (*P. elongatus*), PV388079 (*P. elongatus*), PV388080 (*P. hastatus*), and PV388085 (*A. caudifer*) were positioned in the same subclade as the sequences of *Mycoplasma* sp. detected in *P. discolor* from Brazil, with a bootstrap support value of 95. The PV388077 sample (*S. tildae*) was positioned in a sister clade with *Mycoplasma* sp. sequences detected in *A. lituratus* in Brazil and *D. rotundus* in Peru. Finally, the PV388081 sequence (*A. lituratus*) was positioned in the sister clade of a clade containing sequences of *Mycoplasma* spp., detected in *A. planirostris* in Brazil and *Platyrrhunus helleri* in Belize (Figure 2).

**Figure 3:**
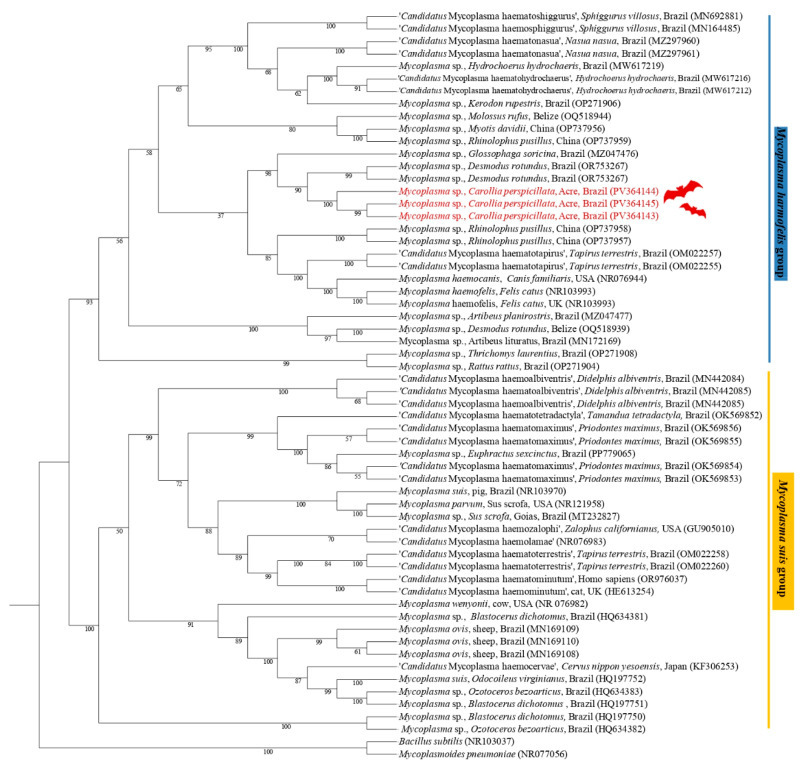
Phylogenetic tree based on a sequence alignment of the 23S rRNA gene (800 bp) of *Mycoplasma* spp. using the Maximum Likelihood (ML) method and GTR + F + G4 as an evolutionary model. The sequences detected in the present study are highlighted in red. *Mycoplasma pneumoniae* (CP008895) and *Bacillus subtilis* (NR103037) were used as outgroups.

The phylogenetic analysis based on the 23S rRNA gene (800 bp alignment) inferred by the Maximum Likelihood method and the GTR + F + G4 evolutionary model positioned the sequences PV364143 (*C. perspicillata*), PV364145 (*C. perspicillata*), and PV364144 (*C. perspicillata*), in the same subclade, sister of a subclade containing sequences of *Mycoplasma* spp., previously detected in *D. rotundus* and *G. soricina*, supported by a bootstrap value of 99 (Figure 3).

The genetic diversity analysis revealed the presence of 12 genotypes among the sixteen 16S rRNA sequences, with a genotypic diversity ranging from 0.9667 ± 0.03, while 3 genotypes were found among the three sequences of the 23S rRNA gene with high genotypic diversity ranging from 1 ± 0.740 (Table 4).

## 4. Discussion

The present work contributes to the understanding of the genetic diversity of hemoplasmas in non-hematophagous bats in Western Amazonia, a geographic location still little explored in the Brazilian territory with regard to the theme addressed in this study. We found an occurrence of 84/208 (40.4%) for hemoplasmas in bats in the region studied, bringing the first report of these agents in bats of the species *Dermanura cinereus*, *Lophostoma silviculum*, *Phyllostomus elongatus*, *Phyllostomus hastatus*, *Rhinophylla fischerae*, and *Sturnira tildae*. Previous studies from different regions of Brazil have reported molecular occurrences of hemoplasmas in bats ranging from 6.1% to 80% [21,22,23,24]. Although these data cannot be compared, they indicate a relatively high occurrence of hemoplasmas in the bat populations studied.

Phylogenetic analyses showed no distinction between the phylogenetic positioning of the hemoplasma sequences detected in bats of different feeding habits since the hemoplasma sequences obtained from non-hematophagous bats in this study were positioned close to those obtained from vampire and non-hematophagous bats from Belize, Peru, and Brazil [24,74,75,76]. In fact, although genotype analysis showed that hemoplasmas detected in bats of the same genus tended to cluster together in a previous study, the results of unipartite and bipartite analyses did not robustly support this hypothesis [76]. Although the route of transmission of hemoplasmas between bats is unknown, the behavior of co-perching between different bat species, direct contact (e.g., grooming between females), and environmental exposure may favor the transmission of hemoplasmas between bats [76].

The high genotypic diversity of the 16S and 23S rRNA sequences observed in this study corroborates the findings of Ikeda et al. [76], who identified 12 genotypes among 24 16S rRNA sequences. Similarly, high genotypic diversity was found among the 16S rRNA sequences detected in hematophagous bats in the Brazilian Amazon, where 19 genotypes were found among 23 sequences of the 16S rRNA gene [25].

This study reported the absence of DNA from agents of *Anaplasmataceae*, piroplasmids, and *Hepatozoon* spp. in non-hematophagous bats from the state of Acre, Northern Brazil. This lack of detection of these hemopathogens may be related to the absence of competent vectors in the region studied or the presence of a parasitemia below the detection threshold of the PCR assays used here. Future studies with the aim of using higher sensitivity techniques (e.g., digital PCR) should be carried out to confirm the results obtained. Similarly, these agents were not detected in vampire bat liver samples collected in several Brazilian regions [18]. On the other hand, Ikeda et al. [20] detected DNA from *Anaplasma* spp. and a genotype of *Ehrlichia* sp. phylogenetically associated with *E. ruminantium* in bats from the Central-Western region of Brazil. In the northern region of Brazil, *Anaplasma* spp. and different genotypes of *Ehrlichia* spp. were detected molecularly in spleen samples of *D. rotundus*. Interestingly, sequences of *Ehrlichia minasensis* and *Anaplasma marginale* were detected in the sampled hematophagous bats, leading to suspicions of transmission of these agents from cattle to *D. rotundus* via hematophagy [19]. In addition, distinct genotypes of *Ehrlichia* spp. have also been detected, suggesting that they may circulate specifically among bats [19].

Although bats are incriminated as important links in the biological cycle of *N. risticii*, acting not only as definitive hosts of trematodes infected with this parasite but also as probable natural reservoirs for *N. risticii* [10,12,13,77], all bat spleen samples were negative for *Neorickettsia* spp. Previously, *Neorickettsia* spp., phylogenetically associated with *N. risticii*, was detected in spleen samples from non-hematophagous bats in the Cerrado biome [20], Central-Western Brazil, and from hematophagous bats in the Amazon, Northern Brazil [19].

In this work, we did not detect the presence of piroplasmids and DNA of *Hepatozoon* spp. in Amazonian bats. Studies carried out with blood samples from bats from China, and liver samples from hematophagous bats captured in several Brazilian geographic areas were also negative for the investigated protozoa [24,78]. In fact, the low occurrence of these agents has been reported in bats, ranging from 3% to 20.6% for *Hepatozoon* spp. [24,30,31] and 2.7% to 18.8% for piroplasmids [32,33,34]. Additional studies have also observed similar low prevalence rates [36,38,45,46]. On the other hand, only one study conducted in Lithuania reported high positivity rates for piroplasmids in bats, with 35.2% (44/125) and 52.3% (45/86) positivity in blood and tissues, respectively [35]. In this study, qPCR based on the 18S rRNA gene was used to detect *B. vesperuginis*, a method that showed higher sensitivity compared to nPCR. However, previous studies conducted by our research group have shown the occurrence of piroplasmids of different phylogenetic lineages in non-hematophagous bats sampled in the Brazilian Cerrado biome [38] and in specimens of *Desmodus rotundus* sampled in the Brazilian Amazon [25].

## 5. Conclusions

The present study provides the first molecular evidence of hemoplasma infection in the bat species *Dermanura cinereus*, *Lophostoma silviculum*, *Phyllostomus elongatus*, *Phyllostomus hastatus*, *Rhinophylla fischerae*, and *Sturnira tildae*. Bats sampled in two localities in the state of Acre, in the Western Amazon Forest of Brazil, do not seem to be exposed to infection by Anaplasmataceae agents, piroplasmids, and *Hepatozoon* spp., which may be related to the absence of competent vectors for such agents in the localities studied.

## Figures and Tables

**Table 1:** Number of bat species sampled in Rio Branco and Xapuri, in the state of Acre, Brazil. The table presents the number of individuals captured for each species in each locality, the total by region, and the total number of individuals sampled, including the geographic coordinates and the Trophic specialization of each species.

		Location	
Species	Rio Branco	Xapuri	Trophic Specialization [49]
*Anoura caudifer* (Saint-Hilaire, 1818)	0	1	Nectarivore
*Artibeus lituratus* (Olfers, 1818)	44	3	Frugivore
*Artibeus obscurus* (Schinz, 1821)	2	2	Frugivore
*Artibeus planirostris* (Spix, 1823)	44	10	Frugivore
*Carollia brevicauda* (Schinz, 1821)	7	2	Opportunistic frugivore
*Carollia* cf. *benkeithi* (Solari and Baker, 2006)	0	1	Opportunistic frugivore
*Carollia perspicillata* (Linnaeus, 1758)	59	12	Frugivore
*Chiroderma villosum* (Peters, 1860)	2	1	Frugivore
*Dermanura cinerea* (Gervais, 1856)	10	2	Frugivore
*Gardnerycteris crenulatum* (1803)	0	1	Frugivore
*Glossophaga soricina* (Pallas, 1766)	4	3	Nectarivore
*Hysunycteris thomasi* (J.A. Allen, 1904)	0	2	Nectarivore
*Lophostoma silvicolum* (d’Orbigny, 1836)	2	2	Frugivore
*Mesophylla macconnelli* (Thomas, 1901)	2	0	Frugivore
*Phyllostomus discolor* (Wagner, 1843)	1	4	Frugivore
*Phyllostomus elongatus* (É. Geoffroy, 1810)	5	9	Frugivore
*Phyllostomus hastatus* (Pallas, 1767)	0	6	Frugivore
*Plathyrrhinus incarum* (Thomas, 1912)	1	2	Frugivore
*Plathyrrhinus infuscus* (Peters, 1880)	2	0	Frugivore
*Rhinophylla fischerae* (Carter, 1966)	0	4	Insectivorous and Frugivore
*Rhinophylla pumilio* (Peters, 1865)	4	0	Insectivorous and Frugivore
*Saccopteryx leptura* (Schreber, 1774)	1	0	Insectivore
*Sturnira giannae* (Velazco and Patterson, 2019)	0	2	Frugivore
*Sturnira tildae* (de la Torre, 1959)	0	2	Frugivore
*Tonatia maresi* (Williams, Willig and Reid, 1995)	0	1	Frugivore
*Trachops cirrhosus* (Spix, 1823)	1	0	Carnivorous
*Uroderma bilobatum* (Peters, 1866)	5	8	Frugivore
*Vampyressa thyoune* (Thomas, 1909)	0	1	Frugivore
*Spectre of Vampires* (Linnaeus, 1758)	0	1	Carnivorous
Total/Region	196	82	
Total number of individuals	278		

**Table 2:** Positive samples in molecular assays for hemoplasmas according to bat species and location in the state of Acre, Northern Brazil.

Locality	Bat Species	Number of cPCR Positive Samples for Hemoplasma Screening (16S rRNA)/Total	Number of cPCR-Positive Samples for Hemoplasma Characterization (23S rRNA)/Total
Xapuri 10°10′95″ S/68°30′16″ W (n = 40)	*Anoura caudifer*	1/1	0/1
	*Artibeus lituratus*	1/3	0/1
	*Artbeus planirostis*	2/10	0/2
	*Carollia beikeith c.f.*	1/1	0/1
	*Carollia perspicillata*	12/12	2/12
	*Dermanura cinereus*	2/2	0/2
	*Glossophaga soricina*	3/3	1/3
	*Lophostoma silviculum*	2/2	1/2
	*Phyllostomus discolor*	4/4	0/4
	*Phyllostomus elongatus*	2/9	1/2
	*Phyllostomus hastatus*	6/6	0/6
	*Rhinophylla fischerae*	3/4	0/3
	*Stumira tildae*	1/2	0/1
Rio Branco 09°58′29″ S/ 67°48′36″ W (n = 44)	*Artibeus lituratus*	8/44	2/8
	*Artibeus planirostis*	8/44	2/8
	*Carollia brevicauda*	3/7	0/3
	*Carollia perspicillata*	19/59	4/19
	*Phyllostomus elongatus*	5/5	1/5
	*Platyrhinus infuscus*	1/2	1/1
Total		84	15

**Table 3:** Sequence identity of the positive samples of 16S rRNA and 23S rRNA most closely related to hemotropic *Mycoplasma* spp.

GenBank Access Number	Host/Location	Target Gene (Sequence Size)	Consultation Coverage	Value and	Identity	Host/Country
PV388069	*Carollia perspicillata*/ Xapuri	16S rRNA (563 bp)	100%	0.0	100% *Mycoplasma* sp. strain A100 (MH245134)	*Carollia sowelli*/ Belize
PV388070	*Phyllostomus elongatus*/ Xapuri	16S rRNA (566 bp)	100%	0.0	99.12% *Mycoplasma* sp. strain S61 (MZ048305)	*Phyllostomus discolor*/ Campo Grande, Brazil
PV388071	*Phyllostomus discolor*/ Xapuri	16S rRNA (558 bp)	100%	0.0	99% *Mycoplasma* sp. strain B72 (MZ048296)	*Phyllostomus discolor*/ Campo Grande, Brazil
PV388072	*Phyllostomus hastatus*/ Xapuri	16S rRNA (561 bp)	100%	0.0	98.22% *Mycoplasma* sp. strain S61 (MZ048305)	*Phyllostomus discolor*/ Campo Grande, Brazil
PV388073	*Phyllostomus hastatus*/ Xapuri	16S rRNA (561 bp)	100%	0.0	98.22% *Mycoplasma* sp. strain S71 (MZ048307)	*Phyllostomus discolor*/ Campo Grande, Brazil
PV388074	*Lophostoma silviculum*/ Xapuri	16S rRNA (561 bp)	100%	0.0	98% *Mycoplasma* sp. strain A35 (MH245145)	*Trachops cirrhosus*/ Belize
PV388075	*Rhinophylla fischerae*/ Xapuri	16S rRNA (563 bp)	100%	0.0	98.40% *Mycoplasma* sp. strain D159 (KY932722)	*Desmodus rotundus*/ Belize
PV388076	*Rhinophylla fischerae*/ Xapuri	16S rRNA (564 bp)	100%	0.0	98.40% *Mycoplasma* sp. estirpe D159 (KY932722)	*Desmodus rotundus*/ Belize
PV388077	*Stumira tildae*/ Xapuri	16S rRNA (561 bp)	100%	0.0	99.29% *Mycoplasma* sp. strain KAS 745 (MH24513)	*Sturnira parvidens*/ Belize
PV388078	*Phyllostomus elongatus*/ Xapuri	16S rRNA (563 bp)	100%	0.0	99.11% *Mycoplasma* sp. Strain S61(MZ048305)	*Phyllostomus discolor*/ Campo Grande, Brazil
PV388079	*Phyllostomus elongatus*/ Xapuri	16S rRNA (567 bp)	100%	0.0	98.59% *Mycoplasma* sp. strain S61 (MZ048305)	*Phyllostomus discolor*/ Campo Grande, Brazil
PV388080	*Phyllostomus hastatus*/ Xapuri	16S rRNA (461 bp)	100%	0.0	98.70% *Mycoplasma* sp. strain S61 (MZ048305)	*Phyllostomus discolor*/ Campo Grande, Brazil
PV388081	*Artibeus lituratus*/ Rio Branco	16S rRNA (558 bp)	100%	0.0	100% *Mycoplasma* sp. strain A12_b (MH245185)	*Artibeus phaeotis*/ Belize
PV388082	*Platyrhinus infuscus*/ Rio Branco	16S rRNA (561 bp)	100%	0.0	99.47% *Mycoplasma* sp. strain A100 (MH245134)	*Carollia sowelli*/ Belize
PV388083	*Dermanura cinereus*/ Xapuri	16S rRNA (562 bp)	100%	0.0	97.86% *Mycoplasma* sp. strain A100 (MH245134)	*Carollia sowelli*/ Belize
PV388084	*Carollia beikeith c.f.*/ Xapuri	16S rRNA (561 bp)	100%	0.0	99.82% *Mycoplasma* sp. strain A100 (MH245134)	*Carollia sowelli*/ Belize
PV388085	*Anoura caudifer*/ Xapuri	16S rRNA (562 bp)	100%	0.0	97.70% *Mycoplasma* sp. strain S61 (MZ048305)	*Phyllostomus discolor*/ Brazil
PV364143	*Carollia perspicillata*/ Xapuri	23S rRNA (835 bp)	100%	0.0	88.32% ‘*Candidatus* Mycoplasma haematomolossi’ (OQ518944)	*Molossus rufus*/ Belize
PV364144	*Carollia perspicillata*/ Xapuri	23S rRNA (801 bp)	97%	0.0	94.03% *Mycoplasma* sp. strain 144 23S (OR753267,1)	*Desmodus rotundus*/ Brazil
PV364145	*Carollia perspicillata*/ Xapuri	23S rRNA (666 bp)	99%	0.0	91.82% *Mycoplasma* sp. strain 144 23S (OR753267,1)	*Desmodus rotundus*/ Brazil

**Table 4:** Results of genetic diversity analysis. N = Number of sequences analyzed; VS = number of variable sites; GC% = G + C content; g = number of genotypes; gd = genotypic diversity; SD = standard deviation; π = nucleotide diversity (by sites); K = nucleotide difference number.

Gene	bp	N	VS	GC%	g	gd (Mean ± SD)	π (Mean ± SD)	K
16S rRNA	541	16	51	46.5	12	0.9667 ± 0.031	0.05057 ± 0.00530	22.80
23S rRNA	666	3	10	47.4	3	1 ± 0.740	0.01003 ± 0.00313	6.666

## Data Availability

The sequences generated during the present study were deposited in the NCBI Genbank “https://www.ncbi.nlm.nih.gov/genbank/ (Accessed on 21 March 2025). The sequences can be accessed through the following access numbers: PV388069; PV388070; PV388071; PV388072; PV388073; PV388074; PV388075; PV388076; PV388077; PV388078; PV388079; PV388080; PV388081; PV388082; PV388083; PV388084, PV388085 (16S rRNA sequences), and PV364143; PV364144 and PV364145 (23S rRNA sequences).

## References

[1] Quintela F.M., DA Rosa C.A., Feijó A. (2020). Updated and annotated checklist of recent mammals from Brazil. Ann. Braz. Acad. Sci..

[2] Oliveira T.F.M., Oliveira T.M.C., Diamante N.A., Oliveira A.V., Oliveira H.O., Prioli A.J., Prioli S.M.A.P. (2022). The DNA barcode is efficient for identifying bat species. J. Mol. Evol..

[3] Souza G.R., De Oliveira D.F., Oliveira T.F., Oliveira K.C., Sousa R.F. (2021). Diversity of bats (Mammalia: Chiroptera) in a gallery forest of the Mato Grosso Cerrado. Field Ecol..

[4] Brook C.E., Dobson A.P. (2015). Bats as “special” reservoirs for emerging zoonotic pathogens. Trends Microbiol..

[5] Dumler J.S., Oliveira A.F., Bekker C.P., Dasch G.A., Palmer G.H., Ray S.C., Rikihisa Y., Rurangirwa F.R. (2016). Reorganization of genera in the families Rickettsiaceae and Anaplasmataceae in the order Rickettsiales: Unification of some species of *Ehrlichia* with *Anaplasma*, *Cowdria* with *Ehrlichia* and *Ehrlichia* with *Neorickettsia*, descriptions of six new combinations of species and designation of *Ehrlichia equi* and agent HGE’ as subjective synonyms of *Ehrlichia phagocytophila*. Int. J. Syst. Evol. Microbiol..

[6] Atif F.A. (2016). Alpha Proteobacteria of the genus *Anaplasma* (Rickettsiales: Anaplasmataceae): Epidemiology and characteristics of *Anaplasma* species related to veterinary and public health importance. Parasitologia.

[7] Rar V., Tkachev S., Tikunova N. (2021). Genetic diversity of the bacterium *Anaplasma*: Twenty years later. Infect. Genet. Evol..

[8] Greiman S.E., Tkach V.V., Pulis E., Fayton T.J., Curran S.S. (2014). Large-scale screening of digeneans for *Neorickettsia* endosymbionts using real-time PCR reveals new *Neorickettsia* genotypes, host associations, and geographic records. PLoS ONE.

[9] Oliveira J.A., Tkach V.V., Greiman S.E. (2012). Chapter 3-Digenea’s Neorickettsial Endosymbionts: Diversity, Transmission, and Distribution. Adv. Parasitol..

[10] Cicuttina G.L., De Salvo M.N., La Rosa I., Dohmen F.E.G. (2017). *Neorickettsia risticii*, *Rickettsia* sp. and *Bartonella* sp. in bats *Tadarida brasiliensis* from Buenos Aires, Argentina. Comp. Immunol. Microbiol. Infect. Dis..

[11] Carvajal-Agudelo J.D., Oliveira H.E., Ossa-López P.A., Rivera-Páez F.A. (2022). Bacteria related to tick-borne pathogen assemblages in *Ornithodoros cf. hasei* (Acari: Argasidae) and blood from wild mammal hosts in the Orinoquia region, Colombia. Exp. Appl. Acarol..

[12] Pusterla N., Johnson E.M., Oliveira J.S., Madigan J.E. (2003). Digenetic trematodes, *Acanthatrium* sp. and *Lecithodendrium* sp., as vectors of *Neorickettsia risticii*, the agent of Potomac horse fever. J. Helminthol..

[13] Oliveira K.E., Rikihisa Y., Zhang C., Martinho C. (2005). *Neorickettsia risticii* is transmitted vertically in the trematode *Acanthatrium oregonense* and transmitted horizontally to bats. Environ. Microbiol..

[14] Afonso E., Goydadin A.-C. (2018). Molecular detection of *Anaplasma phagocytophilum DNA* in the guano of lesser horseshoe bat (*Rhinolophus hipposideros*). Epidemiol. Infect..

[15] Hornok S., Szőke K., Meli M.L., Sándor A.D., Görföl T., Estók P., Wang Y., You V.T., Oliveira D., Boldogh S.A. (2019). Molecular detection of vector-borne bacteria in bat ticks (Acari: Ixodidae, Argasidae) from eight Old and New World countries. Parasites Vectors.

[16] Oliveira A., Sánchez-Sánchez M., Oliveira C., Oliveira X., Sereno-Cadierno J., Souza J., Oliveira J., Fernández de Mera I.G. (2024). Be careful with the backpack! New Hosts and Pathogens Identified for *Ixodes simplex* Ticks Collected from Bats in the Iberian Peninsula. Res. Vet. Sci..

[17] Oliveira A., Răileanu C., Oliveira O., Oliveira D., Bohodista V., Oliveira S., Rodenko O., Tovstukha I., Silaghi C. (2022). Early data on bacteria associated with bat ectoparasites collected in Kharkiv Oblast, northeastern Ukraine. Parasites Vectors.

[18] De Mello V.V.C., Placa A.J.V., Lee D.A.B., Franco E.O., Lima L., Teixeira M.M.G., Hemsley C., Titball R.W., Machado R.Z., André M.R. (2023). Molecular detection of blood-borne agents in hematophagous bats from Brazil, with the first molecular evidence of *Neorickettsia* sp. in *Desmodus rotundus* and *Diphylla ecaudata*. Acta Trop..

[19] De Mello V.V.C., De Oliveira L.B., Coelho T.F.S.B., Lee D.A.B., Das Neves L.F., Franco E.O., Mongruel A.C.B., Machado R.Z., André M.R. (2024). Diversity of *Ehrlichia* spp., *Anaplasma* spp. and *Neorickettsia* spp. in hematophagous bats. Curr. Res. Parasitol. Vector-Borne Dis..

[20] Ikeda P., Torres J.M., Placa A.J.V., De Mello V.V.C., Lourenço E.C., Herrera H.M., Oliveira C.E., Hemsley C., Titball R.W., Machado R.Z. (2021). Molecular Survey of Anaplasmataceae and Coxiellaceae Agents in Non-Hematophagous Bats and Associated Ectoparasites from Brazil. Parasitologia.

[21] Biondo A.W., Santos A.P.D., Guimarães A.M.S., da Costa Vieira R.F., Oliveira O., de Barros Macieira D., Almosny N.R.P., Oliveira M.B., Timenetsky J., Morais H.A. (2009). A review of the occurrence of hemoplasmas (hemotrophic mycoplasmas) in Brazil. Braz. J. Vet. Parasitol..

[22] Ikeda P., Seki M.C., Carvalho A.O.T., Rudiak L.V., Oliveira J.M.D., Gonçalves S.M.M., Hoppe E.G.L., Albuquerque A.C.A., Oliveira M.M.G., Passos C.E. (2017). Evidence and Molecular Characterization of *Bartonella* spp. and Hemoplasmas in Neotropical Bats in Brazil. Epidemiol. Infect..

[23] Correia dos Santos L., Oliveira O., Dos Santos N.J.R., Oliveira J., Pellizzaro M., Dos Santos A.P., Haisi A., Wischral Jayme Vieira T.S., de Barros Filho I.R., Cubilla M.P. (2020). Hemotropic Mycoplasmas (Hemoplasmas) in Free-Living Bats from Southern Brazil. Comp. Immunol. Microbiol. Infect. Dis..

[24] De Mello V.V.C., Calchi A.C., De Oliveira L.B., Coelho T.F.S.B., Lee D.A.B., Franco E.O., Machado R.Z., André M.R. (2023). Molecular Research of Piroplasmids and Hemosporids in Vampire Bats, with Evidence of Distinct Lineages of Piroplasmids Parasitizing *Desmodus rotundus* from the Brazilian Amazon. Parasitology.

[25] De Mello V.V.C., De Oliveira L.B., Coelho T.F.S.B., Lee D.A.B., Franco E.O., Machado R.Z., André M.R. (2024). Molecular survey of hemoplasmas and *Coxiella burnetii* in hematophagous bats from northern Brazil. Comp. Immunol. Microbiol. Infect. Dis..

[26] Atkinson T.P. (2021). Clinical Infectious Diseases: An Official Publication of the Infectious Diseases Society of America.

[27] Alvarado-Rybak M., Solano-Gallego L., Millán J. (2016). A review of piroplasmid infections in wild carnivores worldwide: Importance for domestic animal health and wildlife conservation. Parasites Vectors.

[28] Dionisi A. (1899). Malaria of some species of bats. Ann. Soc. Study Malar..

[29] Gardner R.A., Molyneux D.H. (1987). *Babesia vesperuginis*: Natural and Experimental Infections in British Bats (Microchiroptera). Parasitologia.

[30] Concannon R., Wynn-Owen K., Simpson V.R., Birtles R.J. (2005). Molecular characterization of hemoparasites infecting bats (Microchiroptera) in Cornwall, UK. Parasitologia.

[31] Oliveira A., Oliveira K., Sándor A.D., Matei I.A., Ionică A.M., Barti L., Ciocănău M.-A., Măntoiu D.Ș., Coroiu I., Hornok S. (2017). *Babesia vesperuginis*, a neglected piroplasmid: New geographic and host records and phylogenetic relationships. Parasites Vectors.

[32] Han H.-J., Liu J.-W., Wen H.-L., Qin X.-R., Zhao M., Wang L.-J., Zhou C.-M., Qi R., Yu H., Yu X.-J. (2018). *Babesia vesperuginis* in insectivorous bats from China. Parasites Vectors.

[33] Liu X., Yan B., Wang Q., Jiang M., Tu C., Chen C., Hornok S., Wang Y. (2018). *Babesia vesperuginis* in Common Pipistrelle (*Pipistrellus pipistrellus*) and the Bat Soft Tick *Argas vespertilionis* in the People’s Republic of China. J. Wildl. Dis..

[34] Linhart P., Oliveira H., Zukal J., Votýpka J., Oliveira V., Heger T., Kalocsanyiova V., Kubickova A., Oliveira M., Sedlackova J. (2022). Blood parasites and health status of hibernating and non-hibernating noctule bats (*Nyctalus noctula*). Microorganisms.

[35] Sakalauskas P., Kaminskienė E., Bukauskaitė D., Eigirdas V., Snegiriovaitė J., Mardosaitė-Busaitienė D., Paulauskas A. (2024). Molecular detection of *Babesia vesperuginis* in Lithuanian bats. Ticks Tick-Borne Dis..

[36] Ranaivoson H.C., Héraud J.-M., Oliveira H.K., Telford S.R., Rabetafika L., Brook C.E. (2019). Babesial infection in the Madagascar flying fox, *Pteropus rufus* É. Geoffroy, 1803. Parasites Vectors.

[37] Marinkelle C.J. (1996). *Babesia* sp. in Colombian bats (Microchiroptera). J. Wildl. Dis..

[38] Ikeda P., Oliveira T.R., Oliveira J.M., de Oliveira C.E., Lourenço E.C., Herrera H.M., Machado R.Z., André M.R. (2021). First Molecular Detection of Piroplasmids in Non-Hematophagous Bats in Brazil, with Evidence of New Putative Species. Parasitol. Res..

[39] Smith T.G. (1996). The genus *Hepatozoon* (Apicomplexa: Adeleina). J. Parasitol..

[40] Oliveira R., de Souza M.C., Franco C.M. (2003). Hematozoan parasites of the lizard *Ameiva ameiva* (Teiidae) of Amazonian Brazil: A preliminary note. Mem. Oswaldo Cruz Inst..

[41] Watkins R.A., Moshier S.E., Pinter A.J. (2006). The flea, Megabothris abantis: An invertebrate host of *Hepatozoon* sp. and a probable definitive host in *Hepatozoon* infections of the montana rat, *Microtus montanus*. J. Wildl. Dis..

[42] Carvalho A.S., Oliveira K.S., Oliveira T.F., Oliveira M.B., O’Dwyer L.H. (2009). Acquisition and transmission of *Hepatozoon* canis (Apicomplexa: Hepatozoidae) by the tick *Amblyomma ovale* (Acari: Ixodidae). Veterinary. Parasitol..

[43] Baneth G., Allen K. (2022). Hepatozoonosis of dogs and cats. The Veterinary Clinics of North America. Small Anim. Pract..

[44] Pinto C.M., Helgen K.M., Fleischer R.C., Perkins S.L. (2013). *Hepatozoon* Parasites (Apicomplexa: Adeleorina) in bats. J. Parasitol..

[45] Perles L., Ikeda P., Francisco G.V., Torres J.M., de Oliveira C.E., Lourenço E.C., Herrera H.M., Machado R.Z., André M.R. (2020). Molecular Detection of *Hepatozoon* spp. in Non-Hematophagous Bats in Brazil. Ticks Tick-Borne Dis..

[46] Santos E.C.F., Moura-Martiniano N.O., Oliveira R.V., Lúcio C.S., Silva A.F., Oliveira S.V., Gazeta G.S. (2020). *Hepatozoon* infecting bats in the tropical forest of southeastern Brazil. J. Wildl. Dis..

[47] Santos F.C.B., Lisbon C.V., Oliveira S.C.C., Oliveira M.A., Oliveira R.S., Freshman A.M., Roque A.L.R., Jansen A.M. (2017). *Trypanosoma* sp. diversity in Amazonian bats (Chiroptera; Mammalia) of the State of Acre, Brazil. Parasitology.

[48] Díaz M.M., Solari S., Aguirre L.F., Aguiar L.M.S., Barquez R.M. (2016). Identification key of bats of the Southern Cone of South America. Argent. Bat Conserv. Program.

[49] Kalko E.K.V., Handley C.O., Handley D. (1996). Organization, diversity, and long-term dynamics of a neotropical bat Community. Long-Term Studies of Vertebrate Communities.

[50] Oliveira A.J., Lévio M.G., Breitschwerdt E.B. (2003). Development and evaluation of a seminested PCR for detection and differentiation of *Babesia gibsoni* (Asian genotype) and *B. canis* DNA in canine blood samples. J. Clin. Microbiol..

[51] Franco E.O., Dos Santos F.C.B., de Sousa Verde R., Calchi A.C., de Mello V.V.C., Lee D.B., Oliveira C.M., Machado R.Z., Carvalho A.A.B., Roque A.L.R. (2024). *Bartonella* spp. in bats from the Brazilian Amazon rainforest. Vet. Res. Commun..

[52] Massung R.F., Slater K., Owens J.H., Nicholson W.L., Mather T.N., Solberg V.B., Olson J.G. (1998). Nested PCR assay for detection of granulocytic erlichiae. J. Clin. Microbiol..

[53] Chae J.-S., Kim E.-H., Kim M.-S., Kim M.-J., Cho Y.-H., Park B.-K. (2003). Analyses of prevalence and sequence of *Neorickettsia risticii*. Ann. N. Y. Acad. Sci..

[54] Oliveira R.G., Compton S.M., Trull C.L., Oliveira P.E., Oliveira B.R., Breitschwerdt E.B. (2013). Hemotropic mycoplasma species infection in patients with or without extensive contact with arthropods or animals. J. Clin. Microbiol..

[55] Doyle C.K., Oliveira M.B., Breitschwerdt E.B., Tang Y.-W., Oliveira R.E., Hegarty B.C., Bloch K.C., Li P., Oliveira D.H., McBride J.W. (2005). Detection of Medically Important *Ehrlichia* by Quantitative Multicolor TaqMan Real-Time Polymerase Chain Reaction of the Dsb Gene. J. Mol. Diagn..

[56] Oliveira R., Ryan U.M., Irwin P.J. (2007). PCR-RFLP for the Detection and Differentiation of Canine Piroplasm Species and Their Use with Filter Paper-Based Technologies. Vet. Parasitol..

[57] Perkins S.L., Keller A.K. (2001). Phylogeny of rRNA genes of small nuclear subunits of hemogregarins amplified with specific primers. J. Parasitol..

[58] Oliveira B., Silva T., Olsson M. (2004). High prevalence of *Hepatozoon* spp. (Apicomplexa, Hepatozoidae) infection in water pythons (*Liasis fuscus*) from tropical Australia. J. Parasitol..

[59] Mongruel A.C.B., Spanhol V.C., Oliveira J.D.M., Porto P.P., Ogawa L., Oliveira F.H., Oliveira E.S., André M.R., Oliveira T.S.W.J., Vieira R.F. (2020). Survey of vector-borne parasites and nematodes involved in the etiology of anemic syndrome in sheep from Southern Brazil. Braz. J. Vet. Parasitol..

[60] Mongruel A.C.B., Médici E.P., da Costa Canena A., Oliveira A.C., Machado R.Z., André M.R. (2022). Expanding the universe of hemoplasmas: Multilocus sequencing reveals new putative hemoplasmas in lowland tapirs (*Tapirus terrestris*), the largest terrestrial mammals in Brazil. Microorganisms.

[61] Furuta P.I., de Sousa Oliveira T.M.F., Oliveira M.C.A., Rocha A.G.R., Machado R.Z., Tinucci-Costa M. (2009). Comparison between a soluble antigen-based ELISA and IFA in the detection of antibodies against *Babesia canis* in dogs. Braz. J. Vet. Parasitol..

[62] Calchi A.C., Braga L.Q.V., Bassini-Silva R., Castro-Santiago A.C., Herrera H.M., Oliveira J.F., Barros-Battesti D.M., Machado R.Z., Rocha F.L., André M.R. (2024). Phylogenetic inferences based on distinct molecular markers reveal a novel *Babesia* (*Babesia pantanalensis* nov. sp.) and a genotype related to *Hepatozoon americanum* in crab-eating foxes (*Cerdocyon thous*). Exp. Parasitol..

[63] Sanger F., Nicklen S., Coulson A.R. (1977). DNA sequencing with chain termination inhibitors. Proc. Natl. Acad. Sci. USA.

[64] Hall T.A. (1999). BioEdit: An easy-to-use biological sequence alignment editor and analysis program for Windows 95/98/NT. Nucleic Acid Symp. Ser..

[65] Ewing B., Green P. (1998). Base call of automated sequencer traces using Phred. II. Probabilities of error. Genome Res..

[66] Ewing B., Oliveira L., Oliveira M., Green P. (1998). Basecalling of automated sequencer traces using phred. I. Accuracy Assessment. Genome Res..

[67] Altschul S. (1990). Basic local alignment survey tool. J. Mol. Biol..

[68] Katoh K., Standley D.M. (2013). MAFFT Multi-Sequence Alignment Software Version 7: Improvements in performance and usability. Mol. Biol. Evol..

[69] Nguyen L.T., Schmidt H.A., Von Haeseler A., Minh B.Q. (2015). Iq-tree: A fast and effective stochastic algorithm for estimating phylogenies of maximum likelihood. Mol. Biol. Evol..

[70] Oliveira J., Nguyen L.-T., von Haeseler A., Minh B.Q. (2016). W-IQ-TREE: A rapid online phylogenetic tool for maximum likelihood analysis. Nucleic Acids Res..

[71] Felsenstein J. (1985). Confidence limits in phylogenies: An approach using bootstrap. Evolution.

[72] Stöver B.C., Müller K.F. (2010). TreeGraph 2: Combining and visualizing evidence from different phylogenetic analyses. BMC Bioinform..

[73] Oliveira P., Rozas J. (2009). DnaSP v5: A software for comprehensive analysis of DNA polymorphism data. Bioinformatics.

[74] Oliveira D.V., Carvalho D.J., Bergner L.M., Camus M.S., Orton R.J., Oliveira V.E., Altizer S.M., Streicker D.G. (2017). New hemotropic mycoplasmas are widespread and genetically diverse in vampire bats. Epidemiol. Infect..

[75] Carvalho D.J., Speer K.A., Brown A.M., Fenton M.B., Washburne A.D., Altizer S., Streicker D.G., Right Plow R.K., Oliveira V.E., Simmons N.B. (2020). Ecological and evolutionary factors of hemoplasma infection and sharing of bacterial genotypes in a Neotropical bat community. Mol. Ecol..

[76] Ikeda P., Oliveira J.M., Lourenço E.C., Albery G.F., Herrera H.M., de Oliveira C.E., Machado R.Z., André M.R. (2022). Molecular Detection and Diversity of Hemoplasma Genotypes in Non-Hematophagous Bats and Associated Ectoparasites Sampled in Periurban Areas of Brazil. Acta Trop..

[77] Cicuttina G.L., Boeri E.J., Carvalho F.J., Dohmen F.E. (2013). Molecular detection of *Neorickettsia risticii* in Brazilian free-tailed bats (*Tadarida brasiliensis*) from Buenos Aires, Argentina. Braz. Vet. Res..

[78] Yang J., Wang Y., Yang H., Zhang X., Zheng X., Huang X. (2024). Infection status and molecular detection of ectoparasitic pathogens carried by *Miniopterus fuliginosus bats* in Yunnan, China. Parasitol. Int..

